# Pathogenic role of autoantibodies at the ependyma in autoimmune disorders of the central nervous system

**DOI:** 10.3389/fncel.2023.1257000

**Published:** 2023-09-13

**Authors:** Maxime Bigotte, Adam M. R. Groh, Romain Marignier, Jo Anne Stratton

**Affiliations:** ^1^Department of Neurology and Neurosurgery, Montreal Neurological Institute-Hospital, McGill University, Montreal, QC, Canada; ^2^Forgetting Team—Lyon Neuroscience Research Center, INSERM U1028, CNRS UMR 5292, Claude Bernard Lyon 1 University, Bron, France; ^3^Service de Neurologie, Sclérose en Plaques, Pathologies de la Myéline et Neuroinflammation, Hôpital Neurologique Pierre Wertheimer, Hospices Civils de Lyon, Bron, France

**Keywords:** ependymal cell, neuroinflammation, autoantibodies, B cells, multiple sclerosis, neuromyelitis optica

## Abstract

Ependymal cells make up the epithelial monolayer that lines the brain ventricles and the spinal cord central canal that are filled with cerebrospinal fluid. The ependyma has several functions, including regulating solute exchange between the cerebrospinal fluid and parenchyma, controlling microcirculation of cerebrospinal fluid via coordinated ciliary beating, and acting as a partial barrier. Dysregulation of these functions can lead to waste clearance impairment, cerebrospinal fluid accumulation, hydrocephalus, and more. A role for ependymal cells in a variety of neurological disorders has been proposed, including in neuromyelitis optica and multiple sclerosis, two autoimmune demyelinating diseases of the central nervous system, where periventricular damage is common. What is not known is the mechanisms behind how ependymal cells become dysregulated in these diseases. In neuromyelitis optica, it is well established that autoantibodies directed against Aquaporin-4 are drivers of disease, and it has been shown recently that these autoantibodies can drive ependymal cell dysregulation. We propose a similar mechanism is at play in multiple sclerosis, where autoantibodies targeting a glial cell protein called GlialCAM on ependymal cells are contributing to disease. GlialCAM shares high molecular similarities with the Epstein–Barr virus (EBV) protein EBNA1. EBV has recently been shown to be necessary for multiple sclerosis initiation, yet how EBV mediates pathogenesis, especially in the periventricular area, remains elusive. In this perspective article, we discuss how ependymal cells could be targeted by antibody-related autoimmune mechanisms in autoimmune demyelinating diseases and how this is implicated in ventricular/periventricular pathology.

## 1. Introduction

Modern cellular neuroscience has turned the spotlight on glial cells, and delineated their varied and critical roles in brain function and disease (Duncan et al., 2022). Yet, one glial cell, called the ependymal cell, has yet to be investigated to nearly the same extent as its other glial counterparts. Ependymal cells make up the ependyma, which is a ciliated epithelial barrier that separates the cavities of the brain and spinal cord from the parenchyma. These cavities are filled with cerebrospinal fluid (CSF), and the large majority of ependymal cells play a critical role in regulating CSF micro-circulation via ciliary beating as well as controlling the exchange of solutes between the CSF and brain parenchyma (Del Bigio, 2010). The majority of the ependyma is a partial barrier with high quantities of adherens junctions and some tight junctions (Whish et al., 2015). Sub-portions of the ependyma where circumventricular organs exist, have specialized ependymal cells called tanycytes that have high expression of tight junctions (Mullier et al., 2010). Dysregulation of ependymal cell functions can lead to issues with brain waste clearance and CSF circulation. Indeed, genetic mutations inducing cilia defects in ependymal cells contribute to neurological disorders such as hydrocephalus (Fliegauf et al., 2007). Cilia defects in ependymal cells are also found in the context of bacterial infections (Hirst et al., 2003; Fadaee-Shohada et al., 2010). Ependymal cells incur DNA damage after traumatic brain injury and have been implicated in major neurological disorders like Alzheimer’s disease, where defects could contribute to neurodegeneration if toxic waste clearance and CSF circulation is reduced (Luca et al., 2018). However, for most neurological disorders, the precise triggers of ependymal cell dysfunction remain unclear. In this perspective article, we describe how ependymal cells are altered in two autoimmune diseases, namely neuromyelitis optica (NMO) and multiple sclerosis (MS), and how autoantibodies may contribute to disease pathogenesis in the ependymal/periventricular area.

## 2. Evidence of autoantibody-mediated ependymal alterations in neuromyelitis optica

Neuromyelitis optica is a rare autoimmune disease characterized by inflammatory demyelinating lesions mainly located in the optic nerves and spinal cord, which are associated with visual loss and motor deficits (Wingerchuk et al., 2015). Lesions in periventricular areas, such as in lateral ventricles as well as the third and fourth ventricles, are also common, while the rest of the brain is thought to be spared (Wingerchuk et al., 2015). Initially considered a subtype of MS, a distinction was made 20 years ago due to the discovery that anti-AQP4 immunoglobulin G (IgG) antibodies are consistently present in the sera of the majority (~70–80%) of NMO cases during both active and remission phases of disease (Lennon et al., 2005; Sato et al., 2014). AQP4 is a bidirectional water channel regulating brain water homeostasis that is expressed by astrocytes and ependymal cells (Nielsen et al., 1997; Nagelhus, 2013). Lennon and colleagues found that antibodies from NMO patients bound regions enriched with AQP4 proteins, namely at perivascular astrocytic endfeet, around the brain ventricles, and at the astrocytic subpial glia limitans (Lennon et al., 2005). Interestingly, anti-AQP4 IgGs are rarely found in the CSF of NMO patients, except during active phases of disease, suggesting that the periphery is the main source of anti-AQP4 antibodies (Jarius et al., 2010; Sato et al., 2014). Consistent with this, oligoclonal bands (OCBs) of IgGs are not common in the CSF of NMO patients, and even patients who do test positive for OCBs (27%), lose reactivity within 1–2 years of onset (Bergamaschi et al., 2004). Because inflammatory demyelinating lesions are often found near blood vessels, the scientific community has focused on the pathogenic mechanisms induced by NMO-IgG at astrocytic perivascular foot processes, rather than in other CNS regions. Indeed, the thorough characterization of subpial or periventricular damage in NMO has not been systematically performed (Guo et al., 2017). NMO is considered a primary astrocytopathy, where often complement deposition, immunoglobulins and degranulating neutrophils are at blood vessel-associated lesions (Lucchinetti et al., 2002), and NMO-IgGs are considered the drivers of the formation of these lesions (Takai et al., 2021). However, histopathological analysis shows distinct patterns of NMO lesions in a given patient. For example, some lesions present with high levels of complement, while other lesions have low complement deposition but are enriched with immune cells. Yet, a third subtype presents with neither complement or immune cells but is characterized by dystrophic astrocytes (Misu et al., 2013; Takai et al., 2021). This suggests that different autoantibody-mediated mechanisms are at play. As introduced, AQP4 is also expressed by ependymal cells (Nielsen et al., 1997) and periventricular lesions (abutting the ependyma) are a typical imaging feature of NMO, which is included in the international diagnostic consensus criteria since 2015 (Banker et al., 2012; Wingerchuk et al., 2015). A postmortem brain study has also noted subependymal gliosis and loss of AQP4 immunoreactivity, invasion of granulocytes and microglial activation in the periventricular area (56% of NMO patients, 9/16; Guo et al., 2017). The ependyma in these brains showed reduced AQP4 expression, complement deposition, morphological alterations, and cellular denudation (Guo et al., 2017). Whether these ependyma-associated alterations were a consequence of CSF or parenchymal inflammation, parenchymal damage, or caused by NMO-IgG remains unknown.

Asgari et al. (2013) demonstrated that periventricular alterations occur in mice injected intracerebroventricularly with NMO-IgG purified from plasma. A clear loss of AQP4/GFAP in astrocytes and a disruption in myelin integrity was observed in the periventricular region but no specific analysis on ependymal cells was performed. More recently, high-resolution microscopy and functional assays have been applied to investigate the pathogenic role of NMO-IgG on ependymal cells (Bigotte et al., 2022). Indeed, 24 h of exposure to human NMO-IgGs induced alterations in rat ependymal cells *in vitro* and *ex vivo*, including mis-localization of AQP4 and gap junction protein expression, as well as altered cell morphology. Ependymal cilia function was also altered with motility defects and disorganization of cilia tuft planar polarity. Surprisingly, NMO-IgG increased the expression of proinflammatory cytokines and chemokines expressed by ependymal cells, suggesting that ependymal cells may play a role in immune cell recruitment in NMO. Interestingly, the majority of NMO patients’ IgGs induced ependymal cell alterations (57%, 4/7) but some had no effect even if patients tested positive for anti-AQP4 antibodies. Of note, only one patient out of seven presented with PV lesions based on MRI. It is possible that certain AQP4 epitopes are targeted and cause a disruption in ependymal cells, whereas other epitopes are not. It is also possible that other auto-antibodies drive ependymal dysfunction as auto-immunity is broad in NMO (Ducloyer et al., 2022). Interestingly, immunoglobulins from healthy donors produced a subtle pro-inflammatory ependymal cell profile (less than NMO-IgG but present nonetheless) suggesting that ependymal cells are sensitive to non-NMO-IgGs as well. However, this proinflammatory profile was not associated with defects in ependymal cell function. Although it is clear that ependymal cell alterations occur in response to NMO-IgGs, it is less clear to what extent ependymal cell dysregulation could lead to lesion formation in periventricular regions. When Asgari and colleagues demonstrated periventricular alterations following intracerebroventricular injections of NMO-IgGs, it was done in conjunction with the injection of complement. Importantly, the authors did not observe any effects without this co-injection, suggesting that periventricular alterations occur by complement-dependent mechanisms. This suggests that ependymal cell function could be transiently compromised following exposure to NMO-IgGs but for chronic damage to occur, the complement system may be necessary.

## 3. Proposing a link between autoantibodies and ependymal alterations in multiple sclerosis

Multiple sclerosis is the most common neuroinflammatory autoimmune disorder of the central nervous system (CNS) and is typically characterized by the presence of inflammatory demyelinating lesions. Like in NMO, these lesions can appear in the optic nerve, spinal cord, and periventricular area (but are most consistently around the lateral ventricles compared to other ventricular areas; Adams et al., 1987). Lesions are also well characterized in the cerebellum, subpial areas, and in the white matter throughout the brain (Haider et al., 2014; Fadda et al., 2019). While NMO is mostly relapsing (Wingerchuk et al., 2015), MS is a heterogenous disease in which patients can present with a clinical course characterized by relapsing-remitting phases, a primary progressive course, or progressive course secondary to relapsing-remitting phases (Kuhlmann et al., 2023). Postmortem evaluation of MS patient brain tissue has allowed for the identification of unique lesion patterns, which are independent of symptomatology, suggesting distinct pathogenic mechanisms for each pattern. Pattern I lesions (23%) are laden with T-cells & macrophages, pattern II lesions (56%) have antibodies and complement deposition suggestive of humoral pathology, and pattern III lesions (22%) have oligodendrocyte dystrophy and apoptosis without signs of autoimmune attack (Lucchinetti et al., 2000; Tobin et al., 2021). Patients can present with multiple lesions spreading in time or location but only present with one immunological pattern (Tobin et al., 2021). Such observations raise the possibility that MS is an amalgamation of three distinct disease subtypes, with humoral-mediated disease being the major subtype, reminiscent of NMO (Höftberger et al., 2022).

Indeed, the role of B-cells in the pathological development of MS has recently regained interest (Dendrou et al., 2015). OCBs in CSF are a hallmark feature of MS, and serve as a diagnostic criteria (97% of MS patients are OCB positive; Thompson et al., 2018). While the load of both CSF OCBs of IgGs and IgMs are associated with disease evolution and activity in relapsing–remitting MS (Engel et al., 2020; Hvaring et al., 2022), only OCBs of IgM are associated with progressive MS (Villar et al., 2014; Casanova et al., 2022), suggesting different pathogenic roles of antibodies as disease progresses. Patients with pattern II lesions respond well to plasma exchange, a second line therapy used to remove autoantibodies from the plasma (55% show clinical improvement compared to 30% for pattern I patients; Stork et al., 2018). B-cell-depleting therapies are also highly successful in MS (Comi et al., 2021), and are thought to act on multiple roles of B-cells, such as B-cell/T-cell interactions, cytokine cytotoxic release, and antigen presentation, but also antibody secretion (Comi et al., 2021). Forty percent of MS patients on the anti-CD20 therapy, Rituximab, showed a significant reduction in relapses at 48 weeks post-intervention (Hauser et al., 2008), but the precise mechanisms driving this effect remains unknown. Interestingly, a recent study demonstrated that B-cells in MS are prone to invade the CNS and become antibody secreting only once in the CNS. This study demonstrated that these B-cells are present at the rim of active white matter lesions (Bogers et al., 2023), and their presence is associated with lesion activity (demyelination), intrathecal and tissue levels of MS-IgGs, and the number of resident T4 lymphocytes (Bogers et al., 2023). Interestingly, 88% of pattern I MS patients present with restricted CSF OCBs (i.e., produced in the CSF), suggesting that even with lesion pathology that is suggestive of T-cell and macrophage-mediated pathogenesis, intrathecal humoral-mediated disease mechanisms may also be critical to drive disease (Jarius et al., 2017). In contrast, only 27% of patients with pattern II (and III) lesions have restrictive CSF OCBs, even though lesions are laden with antibodies and complement deposition, suggesting that these patterns of disease may be linked to peripheral sources of autoantibodies (like NMO; Jarius et al., 2017).

Until recently, no MS-specific autoantibodies have been found despite efforts of the research community (Höftberger et al., 2022). However, by interrogating post-translational modifications and single cell analysis of the B-cell repertoire in MS blood and CSF, a recent landmark discovery identified an antibody (IgG) which targets GlialCAM in MS, especially when GlialCAM is post-translationally modified (Lanz et al., 2022). GlialCAM is a cell adhesion molecule involved in regulating cell coupling and gap junctions (Baldwin et al., 2021), and is expressed by most glial cells (except microglia; Favre-Kontula et al., 2008). Interestingly, the focus on GlialCAM as a target in MS occurred due to the remarkable molecular mimicry of this protein with EBV nuclear antigen 1 (EBNA1), which is a peptide derived from the Epstein–Barr virus (EBV) and thought to be necessary, but not sufficient, to cause MS (Aloisi et al., 2023). Other antibodies targeting host antigens that have molecular mimicry with EBV proteins have also been associated with MS, suggesting a polyclonal response (Tengvall et al., 2019). Hence, it is highly likely that a phenomenon of epitope spreading (i.e., when an immune response spreads to other epitopes that are close to the initial target) is occurring with GlialCAM and surrounding transmembrane proteins, and could be part of the oligoclonal IgGs found in MS CSF (Lanz et al., 2023).

GlialCAM is expressed by ependymal cells (Favre-Kontula et al., 2008), and thus could be targeted by autoreactive antibodies present in the blood and CSF of MS patients. Post-translational modifications of GlialCAM expression in ependymal cells are not known but, if present, could facilitate the targeting of ependymal cells by cross-reactivity with anti-EBNA1 antibodies (Lanz et al., 2022). Given the recent discovery that ependymal cells are highly sensitive to anti-AQP4 from NMO patients (Bigotte et al., 2022), it is probable that ependymal cells are also sensitive to antibodies present in MS. Indeed, ependymal cells are altered in MS (Guo et al., 2017; Hatrock et al., 2020) and undergo gliosis, channel dysregulation, and cilia polarity disruption in experimental autoimmune encephalomyelitis (EAE), an animal model used to study MS (Pourabdolhossein et al., 2017). Yet, how ependymal cells become damaged and whether their dysregulation is involved in the emergence and/or progression of periventricular pathology remains unknown. B-cells are found in follicles in the CSF of MS patients, juxtaposed to the subarachnoid space (Magliozzi et al., 2007), and thus could be a source of GlialCAM-binding antibodies that target ependymal cells, which are directly in contact with CSF. This could be especially relevant to MS disease pattern I which is associated with high antibody and intrathecal IgG synthesis in the CSF (Jarius et al., 2017). Given the high incidence of periventricular lesions in MS, it is particularly critical to understand how B-cell mediated ependymal/periventricular damage is governed to elucidate the pathogenesis of MS.

## 4. Pathogenic implications of ependymal alterations in NMO and MS

**Figure 1:**
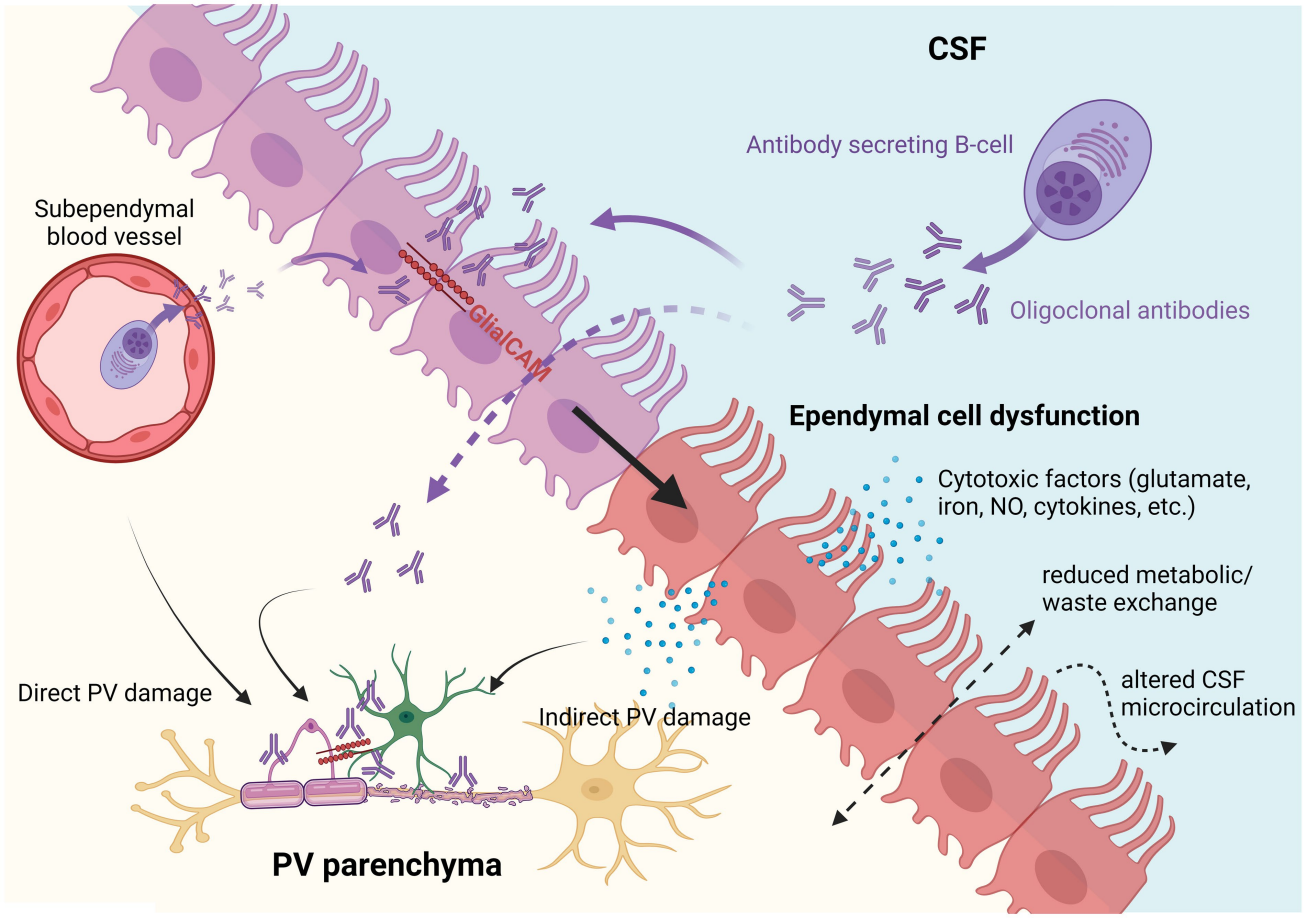
Hypothetical pathogenic implications of multiple sclerosis (MS) autoantibodies on ependymal cells and the periventricular parenchyma. Autoantibodies produced by antibody secreting B-cells either directly in the CSF or from subventricular blood vessels could theoretically induce ependymal cell dysfunction and/or directly target subventricular astrocytes and oligodendrocytes (also expressing GlialCAM). Dysfunctional ependymal cells could lead to indirect periventricular (PV) damage by releasing toxic factors (e.g., glutamate, iron, NO, or proinflammatory cytokines) and by reducing CSF/parenchymal metabolic/waste exchange, and dysregulating CSF microcirculation.

In NMO and MS, the ependymal cell lining is mostly preserved even if small portions are denuded (Guo et al., 2017). Indeed, as shown by Bigotte et al. (2022), the ependymal layer is preserved following NMO-IgG exposure, but ependymal cells become dysfunctional. Dysfunction of ependymal cells can lead to a decreased capacity to exchange nutrients but also to clear toxic waste and adequately propel CSF. Consequently, toxins, pro-inflammatory cytokines, and immune cells could accumulate in the CSF and nearby parenchyma leading to the periventricular pathology observed in both NMO and MS (Figure 1).

A variety of evidence suggests that metabolic balance and toxin clearance functions are dysregulated in ependymal cells in NMO and MS. Aquaporin-4 and connexin-43 alterations have been reported in NMO and could contribute to reduced metabolic support for periventricular cells (Richard et al., 2020; Bigotte et al., 2022). Metal ion transport is also one major and highly conserved transport function orchestrated by ependymal cells (MacDonald et al., 2021). Iron accumulation is toxic and is believed to contribute to neuron and oligodendrocyte death, and to proinflammatory damage in MS lesions (Stankiewicz et al., 2014). It has been shown that ependymal cell alterations can lead to iron accumulation at periventricular sites (Wimmer et al., 2021) and, thus, this mechanism could participate in periventricular lesion development in MS and NMO. In NMO, it has been shown that the excitotoxic release of glutamate by astrocytes targeted by NMO-IgG contributes to neuron and oligodendrocyte death (Marignier et al., 2010, 2016). Ependymal cells also express glutamate transporters, which could be used for release (Del Bigio, 2010). Intriguingly, glutamate injections into rodent lateral ventricles induced ventricular enlargement and damage to periventricular areas (Bai et al., 2022). Thus, as with astrocytes, ependymal cells targeted by autoantibodies could ultimately contribute to adjacent tissue damage by glutamate release. Similarly, the production of pro-inflammatory cytokines and chemokines by ependymal cells could also participate in local tissue damage, the recruitment of immune cells, and an increase in the CSF load of cytokines (Bigotte et al., 2022). Indeed, the presence of proinflammatory cytokines in the CSF has been correlated to the CSF titer of NMO-IgGs and the presence of B-cells in NMO, and to CSF oligoclonal bands in MS (Sato et al., 2014; Farina et al., 2017). In parallel, a reduction in barrier properties could facilitate the passage of toxic or proinflammatory factors, immune cells and autoantibodies present in the CSF directly into the periventricular parenchyma. Finally, ependymal cells could also participate in periventricular pathology by secreting nitric oxide (NO), which is highly toxic and thought to be involved in tissue damage in inflammatory diseases, including MS. Indeed, expression of the inducible nitric oxide synthase (iNOS) has been found in ependymal cells and in MS periventricular lesions (Hill et al., 2004). Radio-clinical images have shown that the volume of the third and the fourth ventricles is increased in NMO and MS (Schneider et al., 2017), and it has been shown by positron emission tomography that CSF flow is reduced in MS (Schubert et al., 2019). CSF flow rate has not been studied in NMO, but it could be associated with the subpopulation of patients with ependymal alterations. These results could implicate ependymal cells. For example, if ependymal cilia fail to propel CSF correctly, this would lead to these outcomes. It is also possible that a lack of proper solute exchange at the ventricular border could also lead to ventricular enlargement as a result of CSF accumulating, as proposed by a recent mathematical model of human CSF dynamics (Yoshida et al., 2022). In any case, if CSF components accumulate adjacent to the ependyma, especially in MS where waste products and cytotoxic factors exist, this could contribute to periventricular damage. Putting all these observations together, it is reasonable to suggest that ependymal cells may play a role in periventricular pathology via multiple mechanisms, including through CSF accumulation; metabolic exchange imbalance with the CSF; as well as direct secretion of toxic factors such as glutamate, NO, and proinflammatory cytokines that are known to induce tissue damage, and which could further recruit immune cells; but further studies are necessary to dissect these mechanisms.

## 5. Conclusion

In this perspective article, we address the specific question of how autoantibodies may contribute to lesion formation in NMO and MS, two diseases where ependymal/periventricular lesions are present. Recent studies evaluating the effects of autoantibodies from NMO patients on ependymal cells suggest a key relationship between autoantibodies and ependymal cells; but how autoantibody-mediated ependymal cell defects contribute to ependymal/periventricular damage remains unclear, as does whether a similar mechanism might be at play in MS. We discuss potential pathogenic mechanisms to explain how autoantibody-mediated ependymal cell defects could contribute to ependymal/periventricular damage, which may be critical for understanding the pathogenic events occurring at CSF borders of CNS autoimmune diseases.

## Author contributions

MB: Conceptualization, Investigation, Writing – original draft. AG: Writing – review & editing. RM: Writing – review & editing. JS: Conceptualization, Funding acquisition, Supervision, Writing – review & editing.

## Funding

The authors declare financial support was received for the research, authorship, and/or publication of this article. This article was funded by the Redpoll Post-Doctoral Fellowship in Neuro Immunology and Neuro Degeneration, Montreal Neurological Institute, McGill University, Montreal (2023), the Canadian Institute of Health Research (486495 and 185656), MS Canada (915179) and Fonds de recherche du Québec en sciences de le santé (296660). AG was supported by a Vanier Canada Graduate Scholarship from the Canadian Institutes of Health Research (2021).

## Conflict of interest

The authors declare that the research was conducted in the absence of any commercial or financial relationships that could be construed as a potential conflict of interest.

## Publisher’s note

All claims expressed in this article are solely those of the authors and do not necessarily represent those of their affiliated organizations, or those of the publisher, the editors and the reviewers. Any product that may be evaluated in this article, or claim that may be made by its manufacturer, is not guaranteed or endorsed by the publisher.

## Abbreviations

AQP4: Aquaporin-4
CSF: Cerebrospinal fluid
CNS: Central nervous system
EAE: Experimental autoimmune encephalomyelitis
EBNA1: Epstein Barr nuclear antigen 1
EBV: Epstein Barr virus
GFAP: Glial fibrillary acidic protein
GlialCAM: Glial cell adhesion molecule
IgG: Immunoglobulin G
iNOS: Inducible nitric oxide synthase
MS: Multiple sclerosis
NMO: Neuromyelitis optica
NO: Nitric oxide
OCB: Oligoclonal band
PV: Periventricular
